# Substrate-Controlled Magnetism: Fe Nanowires on Vicinal Cu Surfaces

**DOI:** 10.3390/nano10010159

**Published:** 2020-01-16

**Authors:** D. Hashemi, M. J. Waters, W. Hergert, J. Kieffer, V. S. Stepanyuk

**Affiliations:** 1 Department of Materials Science and Engineering, University of Michigan, Ann Arbor, MI 48109, USA; mjwaters@umich.edu (M.J.W.); kieffer@umich.edu (J.K.); 2 Institut für Physik, Martin-Luther-Universität, Halle-Wittenberg, Von Seckendorff Platz 1, D-06120 Halle, Germany; wolfram.hergert@physik.uni-halle.de; 3 Max-Planck-Institut für Mikrostrukturphysik, Weinberg 2, D-06120 Halle, Germany; stepanyu@mpi-halle.de

**Keywords:** magnetism, *ab initio*, Monte Carlo simulations, nanowires

## Abstract

Here, we present a novel approach to controlling magnetic interactions between atomic-scale nanowires. Our *ab initio* calculations demonstrate the possibility to tune magnetic properties of Fe nanowires formed on vicinal Cu surfaces. Both intrawire and interwire magnetic exchange parameters are extracted from density functional theory (DFT) calculations. This study suggests that the effective interwire magnetic exchange parameters exhibit Ruderman–Kittel–Kasuya–Yosida-like (RKKY) oscillations as a function of Fe interwire separation. The choice of the vicinal Cu surface offers possibilities for controlling the magnetic coupling. Furthermore, an anisotropic Heisenberg model was used in Monte Carlo simulations to examine the stability of these magnetic configurations at finite temperatures. The predicted critical temperatures of the Fe nanowires on Cu(422) and Cu(533) surfaces are well above room temperature.

## 1. Introduction

The continued need for increasing the information storage content of high-density magnetic recording devices requires the development of new nanostructured magnetic materials such as chains—one-dimensional (1D) periodic linear arrangements of atoms. Most of the experimental methods and potential industrial applications require a high packing density of these chains. 1D periodic linear chains have been investigated experimentally [1,2,3,4,5] and theoretically [6,7,8,9,10,11,12,13,14,15].

Stepped surfaces are common templates for 1D nanostructures [16] since they can take advantage of the 1D symmetry provided by an array of parallel steps on a vicinal surface. Cu surfaces can be prepared with a large number of atom-high steps through a procedure known as step decoration. In this process, the material is deposited on a stepped surface and subsequently nucleates along the edges of the steps with chains or nanostripes growing on the lower terraces along ascending step edges. However, Shen et al. [1,2] demonstrated that Fe nanostripes grow on the upper terraces of stepped Cu(111) surfaces.

**Figure 1:**
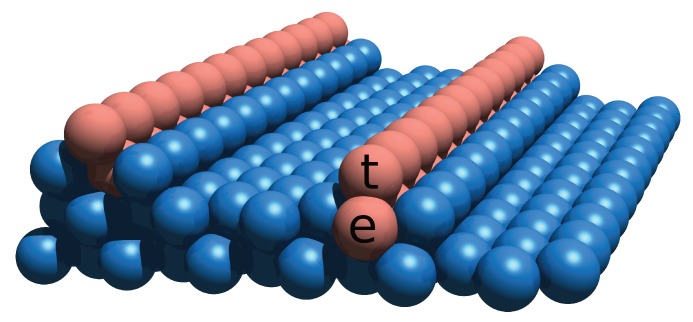
A one-atom-wide Fe double chain (brown) is formed on a vicinal Cu(111) surface (blue). Fe chains at the top (t) and embedded (e) positions are also shown.

In an important study of the growth of linear Fe nanostructures on a stepped Cu(111) surface, Mo et al. [15] examined the elementary diffusion and exchange processes of Fe atoms on the surface by means of *ab initio* calculations based on density functional theory (DFT). This study demonstrated the existence of a special two-stage kinetic pathway leading to the formation of Fe nanowires. In the first stage, Fe adatoms form a very stable 1D atom chain embedded in the Cu substrate behind a row of Cu atoms on the descending step. In the second stage, the embedded Fe chain acts as an attractor for subsequent Fe atoms deposited on the surface, since Fe–Fe bonds are stronger than Fe–Cu bonds. This attraction assists in the formation of a secondary chain of Fe atoms on top of the original embedded Fe chain (cf. Figure 1), resulting in a very stable one-atom-wide iron nanowire formed on the Cu surface. Total energy calculations revealed that the position of the Fe chain at the upper edge is energetically favorable to an Fe chain located at the step edge only if another row of Fe atoms is incorporated underneath the exposed row [15].

In a scanning tunneling microscopy (STM) investigation aided by DFT calculations, Guo et al. [3] confirmed this growth process. A careful study of all atomic processes in the line of Ref. [15] has been used to perform kinetic Monte Carlo (MC) calculations [17]. The simulations demonstrated the growth process as predicted by Mo et al., and have been proven experimentally [3].

The interplay between dimensionality, local environment, and magnetic properties has attracted special interest in such systems. In the following work, a single linear periodic arrangement of atoms is referred to as a chain, while two parallel chains, either isolated or embedded in the Cu(111) surface, are called a wire.

The present investigation provides a systematic discussion of the magnetic properties of 1D Fe nanostructures grown on a vicinal Cu(111) surface using the above-mentioned template (cf. Figure 1). Detailed information on the real structures and magnetic states of such systems is given in Ref. [18,19]. Ferromagnetic ordering is achieved for Fe wires deposited on this template. We present a systematic investigation of the magnetic couplings for Fe embedded in the Cu surface with terraces ranging from three to eight lattice constants wide. The analysis of the exchange coupling and of the magneto-crystalline anisotropy allows us to set up a classical Heisenberg model to study finite temperature effects.

The outline of this paper is as follows. Section 2 is devoted to a brief description of the theoretical framework and setup that we have used. Exchange parameters extracted from the DFT calculations are discussed in Section 3.1.2. The magnetic phase transition to the paramagnetic state and adequate estimation of the critical temperature on the basis of numerical simulations are discussed in Section 3.2.2. Finally, in Section 4 we summarize our main results and conclude.

## 2. Theoretical Method

The calculations were performed within the framework of spin-polarized density functional theory, using the Vienna *ab initio* simulation package (VASP) [20,21]. The frozen-core full-potential projector-augmented-wave (PAW) method was used [22], applying the generalized gradient approximation of Perdew, Burke, and Ernzerhof (GGA-PBE) [23].

The computational details and convergence checks are the same as those in our previous study [18], with minor changes that are explained in Section 3.

A supercell containing twelve Cu layers, corresponding to between 72 and 192 Cu atoms for Cu(*n* + 2,*n*,*n*), with *n* = 2–7, was constructed to model the Cu(111) stepped surface. Conversely, the terraces ranged from three to eight lattice constants wide. The distance from one slab to its nearest image was equivalent to 13.5 Å. The number of k points was chosen according to the requirement of the number of atoms times the number of k points in the irreducible Brillouin zone.

## 3. Results and Discussion

### 3.1. Results from Previous Works

#### 3.1.1. Real Structure of Embedded Fe Wires

We compared the relaxation of a Cu(111) surface with an embedded Fe chain with that of a clean Cu(111) surface. The extent of relaxation in the second subsurface layer is generally small. The relaxation in the first subsurface layer is larger but only significantly so at the step edge. The relaxation of the surface layer of Cu(111) is dominated by lateral and inward relaxations. Lateral relaxation is directed towards the center of the terrace, causing compression. The lateral relaxation in the middle of the terrace is small. In general, the surface layer shows an inward relaxation, which is large at the step edge. Together with the outward relaxation for the first Cu atom of the terrace, the relaxations reduce the interatomic distances at the step edge. Significantly larger relaxation is observed when one row of Cu atoms is substituted by one row of Fe atoms behind the step edge. From a structural point of view, the Fe chain acts as a “center of attraction.” On clean Cu(111) surfaces, in the center of a terrace, practically no lateral shift can be seen, whereas a Cu atom at the same site will shift towards the embedded Fe chain. The Cu atoms at the step edge are also strongly attracted to the Fe chain. The inward relaxation of the Fe chain is much larger than the corresponding relaxation of a Cu atom at this site. In summary, the Fe chain dramatically increases the tendency for compression near the step. The predominant structural reorganization is an inward relaxation of the Fe atoms relative to their ideal positions. For the Fe wire, the inward relaxations of Fe atoms at the top and embedded positions are 22.5% and 5.9%, respectively, relative to the Cu lattice plane distance. The Fe–Fe bond length is 2.58 Å between the embedded Fe atoms; however, it is 2.28 Å between the Fe atoms at the top and embedded positions. The latter is only about 2% larger than the corresponding Fe–Fe bond length in the isolated Fe zigzag chain [8].

#### 3.1.2. Magnetic Exchange Interactions

The analysis of the exchange couplings and the magneto-crystalline anisotropy allows the setting up of a classical Heisenberg model to study finite temperature effects in Section 3.2.2.

**Figure 2:**
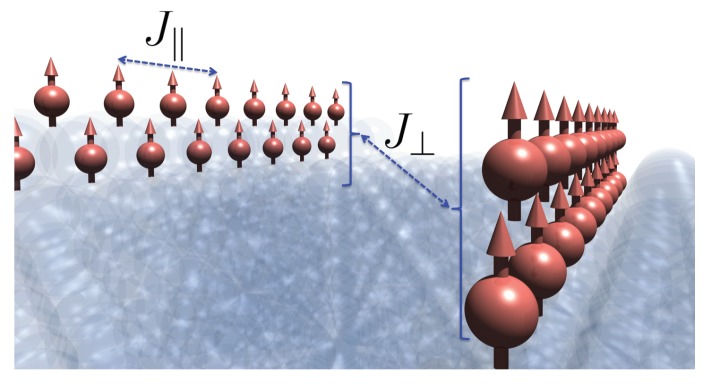
Schematic diagram showing the magnetic interactions of Fe wires. $J_{\|}$: Intrawire exchange coupling, and $J_{⊥}$: Interwire exchange coupling.

For the Fe wires grown on vicinal Cu(111) surfaces, the absolute magnetic moments of Fe atoms at the top and embedded positions are 2.41 and 2.94 $\mu_{B}$, respectively [18]. There are two magnetic interactions between these moments: The intrawire ($J_{\|}$) and interwire ($J_{⊥}$) magnetic couplings (as shown in Figure 2), both of which will be explored in this study.

The Heisenberg theory of magnetism maps magnetic interactions in a material onto localized spin moments. The resulting classical Hamiltonian

(1) $$H=-\sum_{i\neq j}J_{ij}e_{i}\cdot e_{j}-\sum_{i}K_{i}{\left(e_{i}\cdot e_{K}\right)}^{2}$$

contains the unit vectors $e_{i(j)}$ of the magnetic moments, the exchange parameters $J_{ij}$, the magnetic anisotropy energy (MAE) $K_{i}$ (at site *i*), and the unit vector along the magnetization easy axis $e_{K}$. Here, *i* and *j* index the sites.

The MAE is 4.76 meV per site [24]. Using a constant value for MAE does not have any effect on the calculation of exchange couplings since our previous study showed that the MAEs of Fe and the embedded Fe sublattices are equal [24].

$J_{ij}$ can be calculated by making parallel and antiparallel alignments of the moments. Therefore,

(2) $$J_{ij}=\frac{H_{AF}-H_{FM}}{2},$$

where $H_{AF}$ and $H_{FM}$ are the DFT total energies calculated for antiparallel alignment of the moments and parallel alignment of the moments, respectively.

The interwire coupling constants are calculated in a similar manner by exploiting supercells doubled in the direction perpendicular to the wires, and for parallel and antiparallel alignments of the moments on the two wires on each side of the supercell.

#### 3.1.3. $J_{\|}$: Intrawire Exchange Coupling

In order to systematically study the intrawire exchange coupling of Fe wires, three systems were investigated: Freestanding Fe chains, freestanding Fe wires, and embedded Fe wires. In freestanding Fe chains, the atomic distances were constrained to the Cu bond length of the Cu(111) substrate in order to simulate a freestanding equivalent to a singular Fe chain in the Fe wire on the substrate. A freestanding wire was studied as an equivalent to the one embedded into the Cu(111) surface. All interatomic distances also correspond to the Cu bond length of the substrate in this case.

A central task for mapping onto a classical Heisenberg model is the determination of exchange constants and magneto-crystalline anisotropy. The exchange constants can be extracted from DFT calculations either by comparing the total energies of several artificial collinear magnetic structures or by applying the magnetic force theorem in the framework of the Korringa–Kohn–Rostoker (KKR) Green’s function method [25,26]. In addition to these two methods, artificial noncollinear structures can be used to study exchange interactions in the Heisenberg model by choosing noncollinear states that can be controllably switched on and off. Noncollinear configurations used to calculate the exchange parameters for free-standing and embedded wires are given in Refs. [27,28].

*Ab initio* investigations of the freestanding Fe wire revealed that nearest-neighbor-exchange interactions dominate [27,28]. Next-nearest-neighbor interactions are an order of magnitude smaller than nearest-neighbor interactions; therefore, we restrict the Heisenberg model to nearest-neighbor interactions only. Exchange constants $J_{ij}$ can be extracted from DFT calculations by comparing the total energies of several artificial noncollinear magnetic structures. In this approach, we selectively switch on or off interactions between atoms *i* and *j* by deliberately choosing those noncollinear states.

**Figure 3:**
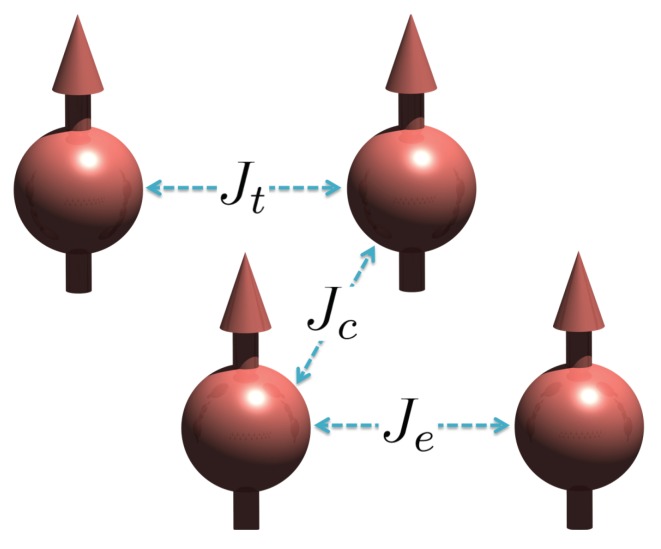
Schematic diagram showing that the Fe wires have the ferromagnetic ground state and the magnetic interactions within the Fe wires: $J_{t}$, $J_{c}$, and $J_{e}$.

$J_{\|}$ can be broken down into three main couplings (as shown in Figure 3): Magnetic couplings between Fe atoms at the top position, $J_{t}$, crossing magnetic couplings between the Fe chain at the top position and the embedded Fe chain, $J_{c}$, and magnetic coupling between embedded Fe atoms, $J_{e}$.

Noncollinear configurations used for calculation of the exchange parameters for freestanding Fe chains and freestanding and embedded Fe wires can be found in Ref. [27]. The obtained exchange parameters published in Ref. [27] for Fe systems are summarized in Table 1. As depicted in Figure 3, the top and the embedded Fe moments are ferromagnetically ordered [18].

Our calculations show that the magnetic moments are constant for the different noncollinear configurations within a specific system, and symmetrically equivalent arrangements lead to the same exchange parameters. These data suggest that the exchange constant for free-standing Fe chains is consistent with the literature [8,9,18]. The magnetic ground state for free-standing Fe chains is in agreement with the results in Ref. [8] for the relaxed chain, since the relaxed bond length in the ferromagnetic state is close to the Cu bond length.

The calculated exchange constants show that Fe wires have ferromagnetic ground states and relaxation effects that are seen in the exchange constants of the embedded systems. The exchange constants in Fe wires are generally smaller than in corresponding linear Fe chains due to an increased coordination number in the planar equilateral triangle ribbon form of Fe wires. The stronger hybridization due to the inward relaxation of the Fe wires leads to smaller intrawire exchange constants.

### 3.2. Results from This Work

#### 3.2.1. $J_{⊥}$: Interwire Exchange Coupling

**Figure 4:**
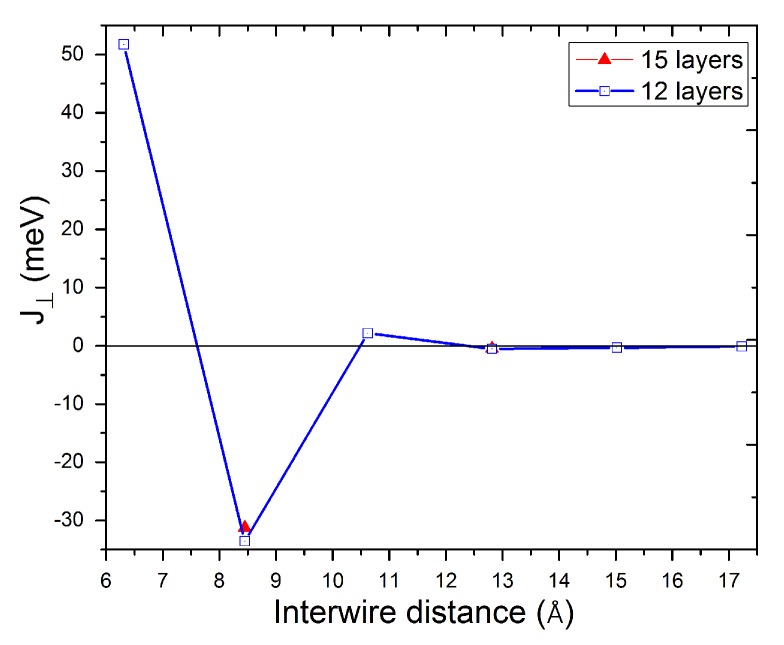
The interwire coupling constants of Fe wires as functions of interwire separation. Blue square and red triangle symbols represent the couplings calculated using 12 and 15 layer slabs, respectively. The blue line connecting the symbols is a guide to the eye.

It is known that surface-state electrons on the (111) surfaces of noble metals create two-dimensional (2D) nearly free electron gases which are confined to top layers at the surfaces. Electrons in these states move along the surface, causing scattering of the surface electrons by nanostructures formed on the surface. This scattering leads to quantum interference patterns in the local density of states (LDOS) and long-range oscillatory interactions between adsorbates [29]. In previous studies, the long-range interactions have been attributed to the surface states of Cu(111) surfaces [30,31]. Based on the calculations presented in Ref. [32], we concluded that only considering 18 layer slabs of Cu produces a band structure that is comparable to experimental energy dispersion. Here, the interwire coupling constants are determined by making parallel and antiparallel alignments of the moments in the Fe wires: Energy differences are not calculated according to the absolute value of energy alone. Our calculations showed that the interwire couplings (energy differences) converge faster than the absolute values of energies. These couplings were converged for slabs as thin as 12 layers. As one can see in Figure 4, using a 15 or 12 layer slab of Cu gives a negligible difference for the interwire couplings obtained for two interwire separations. Therefore, a 12 layer slab of Cu was used to simulate the Cu(111) surface and its surface states. A direct relaxation calculation for such a big system is very expensive; therefore, the four top-most relaxed layers of an eight-layer slab of Cu(111) were taken and replaced on the corresponding geometry of a twelve-layer slab of Cu(111) surface, mimicking the relaxed geometry, while the eight remaining bottom layers were fixed in their ideal bulk positions. The strength of the interwire magnetic coupling can be deduced from the energy difference of the ferromagnetic and antiferromagnetic oriented wires, with supercells doubled in a direction perpendicular to the wires.

To construct a Heisenberg Hamiltonian which takes into account all of the magnetic interactions, $J_{\|}$, effective intrawire couplings, and $J_{⊥}$, the interwire couplings are required. The effective intrawire couplings were determined in Section 3.1.3. Now, we discuss how to estimate the interwire couplings. In principle, there are three interchain coupling constants in the cell. The first is the coupling between the embedded Fe chain and embedded Fe chain in the nearest neighboring wire. The second is the coupling between the embedded Fe chain and the deposited Fe chain at the top position in the nearest neighboring wire. Finally, there is the coupling between the deposited Fe chain on top and the deposited Fe chain at the top position in the nearest neighboring wire. These calculations are computationally demanding due to possible magnetic configurations besides purely ferromagnetic and antiferromagnetic configurations. These extra configurations are necessary as correction factors for exchange couplings even though they may not be energetically favored. The difference between the calculated exchange interactions may be too small, which begs the question of whether this difference has a significant effect on the estimated transition temperature. Therefore, the current study has been confined to effective interwire coupling constants. The interwire coupling constants were estimated using Equation (2) by making parallel and antiparallel alignments of the moments on the two wires on each side of the supercell.

The calculated exchange couplings as functions of interwire separation are shown in Figure 4 and summarized in Table 2. The exchange constants reflect the result that wires have a ferromagnetic ground state on Cu(422). However, there is a strong antiferromagnetic coupling for the nanowires on Cu(533). On Cu(644), the coupling becomes weakly ferromagnetic again. However, on higher-index Cu(111) vicinal surfaces, we observe weak antiferromagnetic ordering. Relaxation effects are insignificant for the interwire couplings.

Similarly to the current study, Ruderman–Kittel–Kasuya–Yosida-like (RKKY) interactions have been observed not only in metallic layered systems, but also between magnetic nanostructures deposited on metal surfaces in which the magnetic interactions are often mediated by surface-state electrons [33,34,35,36].

A rough estimation of the envelope of the magnitude of $J_{⊥}$ suggests an asymptotic decay with the inverse square of the interwire separation. This is in agreement with the *ab initio* calculations and STM experiments that predicted similar interactions between 3*d* magnetic nanostructures on Cu(111) surfaces caused by surface-state electrons [35,37,38]. It is worth noting that the Cu bulk states can affect the interaction energies at relatively short interwire separations because the Fe wires couple to the Cu bulk bands as well. The magnetic interaction energy in the bulk asymptotically decays with the inverse fifth power of the interatomic distance [39].

Long-range interactions between the nanostructures on vicinal Cu(111) surfaces are distinct from those on Cu(111) surface for two reasons: First, the surface states are affected by the electronic potential at the steps which are close to the Fermi energy in vicinal Cu(111) surfaces. Second, the Fe wires significantly affect the surface states on vicinal surfaces [40].

#### 3.2.2. Magnetism at Finite Temperatures

It is necessary to calculate some well-defined macroscopic property, which ensures the correct implementation of interactions in a system. The critical temperature ($T_{c}$) of the investigated systems was determined using MC simulations. $T_{c}$ of a nanowire is primarily determined by the strength of the exchange interaction between spins and the magnetic anisotropy energies. For the representation of the interwire interaction, $J_{⊥}$ is used as given in Table 2.

During MC simulations, lattices with 40 × 40, 42 × 42, and 44 × 44 unit cells with four atoms per unit cell were used. The MAEs of the first row of the Fe sublattices are equal [24]. Periodic boundary conditions were applied in the direction of and perpendicular to the wires. The critical temperature of the system was found by relaxing into thermodynamical equilibrium with 10,000 MC steps per temperature step. Correlation effects were accounted for by averaging over 15,000 measurements, between each of which were two MC steps. To improve the statistics, the averaging of over 20 temperature loops was performed and importance sampling was performed using the Metropolis algorithm. $T_{c}$s were determined using the specific heat and, in the case of a ferromagnetic system, the susceptibility χ and 4th-order cumulant $U_{4}$.

**Figure 5:**
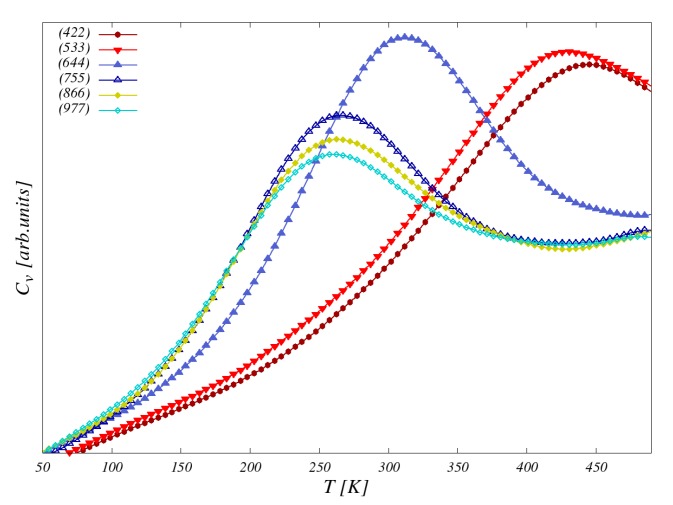
Heat capacity of the Fe nanowires for the magnetic anisotropy energy (MAE) of 4.76 meV.

Figure 5 shows the specific heats for an Fe wire on the vicinal Cu(111) surfaces. We observe that there is a $T_{c}$ for all systems. In our previous study [27], we observed no magnetic ordering for a vanishing MAE, in agreement with the Mermin–Wagner theorem [41]. Our results also show that an increase in interwire couplings stabilizes the moments against thermal fluctuation and, thus, leads to an increase of the $T_{c}$. Furthermore, the phase transition broadens as the interwire couplings are increased. The calculated $T_{c}$s are below room temperature and close to each other for Fe wires on Cu(977), Cu(866), and Cu(755). The interwire couplings $J_{⊥}$ are very small and the $T_{c}$s for these systems are determined by their intrawire couplings, which are the interactions responsible for the ferromagnetic ordering within the Fe wires. The $T_{c}$ is close to room temperature for the Fe wire on Cu(644) and well above room temperature for Cu(533) and C(422). This can be traced back to the high values of $J_{⊥}$ for these systems. A summary of the $T_{c}$s of all systems for MAE = 4.76 meV can be found in Table 3.

## 4. Conclusions

*Ab initio* DFT calculations were used to set up a classical anisotropic Heisenberg model to study the finite temperature properties of Fe wires embedded in Cu(111). To do this, exchange parameters, including intrawire and interwire couplings, were extracted from non-collinear and collinear DFT calculations, respectively. The intrawire couplings were one order of magnitude higher than the interwire couplings for relatively large interwire separation. A slab of Cu of at least 12 layers thick was used to simulate the Cu(111) surface states to provide converged values for the interwire couplings. The interwire exchange couplings of the Fe wires across the vicinal Cu(111) surface oscillated with the interwire separation. This provides a reliable means for stabilizing the magnetic ordering of the Fe nanowires in either a ferromagnetic or an antiferromagnetic configuration.

This study provides a technologically feasible way of tailoring 1D magnetic nanostructures adsorbed on vicinal Cu(111) surfaces. The critical temperatures of the systems with shorter interwire separation are well above room temperature. This is a strong indication that these nanowires have potential applications in high-density magnetic data storage.

## Figures and Tables

**Table 1:** Exchange constants for the freestanding Fe chain, freestanding Fe wires, and the embedded Fe wires. The definition of the constants in the Heisenberg model incorporates the magnetic spin moments. All values are given in meV.

***freestanding Fe chain***	
*J*	114.54
***freestanding Fe wire***	
$J_{t}$	80.33
$J_{c}$	150.42
$J_{e}$	80.33
***embedded Fe wire***	
$J_{t}$	79.76
$J_{c}$	80.00
$J_{e}$	74.58

**Table 2:** Exchange coupling constants ($J_{⊥}$) of Fe wires on Cu(111) stepped surfaces.

**Surface**	(4,2,2)	(5,3,3)	(6,4,4)	(7,5,5)	(8,6,6)	(9,7,7)
**Seperation (Å)**	6.31	8.45	10.62	12.81	15.02	17.23
$J_{⊥}$ **(meV)**	+51.75	−33.52	+2.16	−0.55	−0.32	−0.14

**Table 3:** Critical temperatures ($T_{c}$) of Fe wires on the vicinal Cu(111) surfaces for the MAE of 4.76 meV. The temperature values are given in Kelvin.

**Surface**	(4,2,2)	(5,3,3)	(6,4,4)	(7,5,5)	(8,6,6)	(9,7,7)
$T_{c}$	445	431	312	271	266	262
